# Incidence and risk factors for musculoskeletal adverse effects associated with daptomycin in patients receiving outpatient parenteral antimicrobial therapy

**DOI:** 10.1017/ash.2025.10087

**Published:** 2025-07-31

**Authors:** Meaghen B. Wiley, Kiya K. Bennett, Emily A. Siegrist, Stephen B. Neely, Joseph Sassine, Bryan P. White

**Affiliations:** 1 University of Oklahoma College of Pharmacy, Oklahoma City, OK, USA; 2 OU Health, Oklahoma City, OK, USA; 3 Department of Medicine, University of Oklahoma College of Medicine, Oklahoma City, OK, USA

## Abstract

**Background:**

Daptomycin is preferred in outpatient parenteral antimicrobial therapy (OPAT) due to daily dosing. Elevations in creatine phosphokinase (CPK) of 3%–10% and musculoskeletal adverse events have been described with daptomycin, but data regarding risk factors and frequency of monitoring in the OPAT setting is limited. We evaluated the incidence and risk factors for CPK elevation and musculoskeletal adverse effects in patients receiving daptomycin OPAT.

**Methods:**

This was a single-center, retrospective cohort study of adults on OPAT with daptomycin and at least two CPK values. The primary outcome was the incidence of CPK values greater than 500 U/L.

**Results:**

We included 127 patients. Most patients were male (55.1%), and the median age was 56 years (IQR 46–63). The most common indication was bone/joint infections (73.2%, n = 93). The median daptomycin dose was 7.4 mg/kg/day (IQR 6.1–8.1) and duration of therapy was 37 days (IQR 21–44). Fifteen patients (11.8%) experienced a CPK greater than 500 U/L within a median 13 days (IQR 9–16). Five patients (3.9%) developed rhabdomyolysis. Independent predictors of CPK>500 U/L included male sex (OR, 4.2 [95% CI, 1.05–16.61]; *P* = .0424) and cerebrovascular disease (OR, 11 [95% CI, 1.21–99.86]; *P* = .0332).

**Conclusions:**

The incidence of CPK elevation was similar previously reported rates. This expands the literature to patients with daptomycin doses>6 mg/kg and prolonged durations of therapy. The incidence of CPK elevation and time to onset of 9–16 days supports the current recommendations for weekly lab monitoring.

## Introduction

Daptomycin is a cyclic lipopeptide with activity against gram-positive bacteria, including methicillin-resistant *Staphylococcus aureus* (MRSA) and vancomycin-resistant *Enterococcus* spp. (VRE).^1^ Daptomycin was initially approved for doses of 4–6 mg/kg once daily.^1^ However higher daptomycin doses (≥8 mg/kg) are often required to achieve adequate bactericidal activity and prevent resistance development and treatment failure.^2,3^ Severe infections warranting treatment with daptomycin often require prolonged durations of 4–12 weeks.^2,4–6^ To decrease the hospital length of stay and improve patient outcomes, outpatient parenteral antimicrobial therapy (OPAT) services are used in clinical practice.^7–9^ Literature shows that OPAT patients tend to prefer antibiotics with once daily dosing over multiple infusions per day, as well as antibiotics that do not require frequent dose adjustments for drug serum concentrations.^8–10^ Nevertheless, daptomycin is known to cause creatine phosphokinase (CPK) elevation at rates of 3%–14%^6,11–15^ and musculoskeletal adverse effects (ie, myopathy and rhabdomyolysis).^1,7^ The rate of rhabdomyolysis and myopathy is lower at less than 1%^13^ and 0%–4%,^11,12,15,16^ respectively. Although the level of CPK elevation as a predictive marker for musculoskeletal events has not been determined in the literature, an increased incidence of musculoskeletal events has been described with higher daptomycin doses for longer treatment courses.^2,4–6^ With dose and duration both being associated with an increased incidence of musculoskeletal events, it is unclear whether patients on both higher doses and longer durations of daptomycin with OPAT would have different risk factors.

Therefore, the 2018 Infectious Diseases Society of America (IDSA) guideline for the management of OPAT recommends weekly CPK monitoring for all patients receiving daptomycin.^7^ However, factors such as concomitant use of a statin or antihistamine, body mass index (BMI)>30 kg/m^2^, higher baseline CPK, and impaired renal function have been associated with elevated CPK during treatment with daptomycin.^17,18^ There is a lack of data exploring the incidence of and risk factors for the development of musculoskeletal adverse effects in patients receiving daptomycin through OPAT, and subsequently the appropriate CPK monitoring interval in these patients. Therefore, the purpose of this study is to evaluate the rate and risk factors for CPK elevation and musculoskeletal adverse effects associated with daptomycin in patients receiving OPAT.

## Methods

### Study design and population

This single-center, retrospective, Institutional Review Board-approved, cohort study evaluated patients who received outpatient antibiotic monitoring for daptomycin by the Home Antibiotic Pharmacy Consult Program^19^ from July 1, 2016, to September 30, 2022, at the University of Oklahoma Medical Center. Patients were identified from a database of consult orders for OPAT, and those 18 years or older with at least two documented CPK laboratory values were included for evaluation. For patients with multiple daptomycin OPAT consults, only the initial consult order was included for evaluation. During the study period, patients with obesity on daptomycin were dosed using adjusted body weight.^20^ Standard weekly monitoring of CBC/CMP/CK was performed for patients on daptomycin. When abnormal labs were received, the OPAT pharmacist would reach out to the patient to determine what symptoms were present, discuss a plan with an ID physician, and communicate the plan to the patient.

### Data collection

Data were collected via the inpatient electronic health record (EHR), Meditech, the outpatient EHR, Centricity, and the home antibiotic pharmacy consult database. The following data was collected: name, age, sex, actual body weight (most recent prior to treatment initiation), height, body mass index (BMI), social history, comorbid conditions, concurrent medications, weekly lab results including creatine phosphokinase (CPK—considered baseline if obtained within seven days of therapy initiation), OPAT treatment plan, reported adverse events (myopathy or myalgia, rhabdomyolysis), and hospital admission due to a daptomycin-related adverse event. Creatinine clearance was calculated using height, weight, and creatinine.^21^

### Study outcomes

The primary outcome was the incidence of CPK elevation greater than 500 U/L. Secondary outcomes included CPK elevation greater than 1,000 U/L or greater than 2,000 U/L^7^, the development of myopathy, myalgia, or rhabdomyolysis, discontinuation of daptomycin therapy due to concern for a daptomycin-related adverse event, hospital admission secondary to a confirmed or suspected adverse event from daptomycin therapy, and incidence of complications of daptomycin as a composite of CPK greater than 2,000 U/L, myopathy or myalgia, rhabdomyolysis, discontinuation of daptomycin therapy, or hospital admission for a daptomycin-related adverse event. These secondary outcomes were also assessed in time-to-event analyses. Myopathy or myalgia and rhabdomyolysis were identified based on physician diagnosis and documentation in the medical chart. Additionally, we examined potential risk factors for primary and secondary outcomes using patient demographic and clinical characteristics listed under data collection. Factors evaluated included age, sex, social history, comorbid conditions, concurrent medications, baseline CPK and CrCl, mg/kg dose, and treatment duration.

### Statistical analysis

Data were summarized using descriptive statistics. Categorical variables were reported as a frequency (percentage) and analyzed using χ^2^ or Fisher’s Exact tests. Continuous variables were reported as a median (interquartile range) and analyzed using Wilcoxon Two-Sample test. Musculoskeletal adverse events, therapy discontinuation, and hospital readmission were analyzed as those who experienced the primary outcome (CPK > 500) and those who did not. Logistic regression was used to examine adjusted associations between our primary outcome (CPK > 500) with demographic and clinical characteristics. Initial variables included in the model were daptomycin dose, SCr, CPK, BMI > 30, social history of alcohol, and *Methicillin-resistant Staphylococcus epidermidis* (MRSE). An additional exploratory model with backward selection for inclusion if *P* < .10 was used to determine potential associations included the aforementioned variables along with sex, cerebrovascular disease, diabetes mellitus, hypothyroidism, cancer with active treatment, any statin use, any antihistamine use, E. faecalis, and osteomyelitis, SSTI, complicated bacteremia, or prosthetic joint infections. Unadjusted and adjusted odds ratios with 95% confidence intervals are reported. Alpha was set at .05 and SAS version 9.4 was used for all analyses.

## Results

### Baseline characteristics

**Figure 1.**
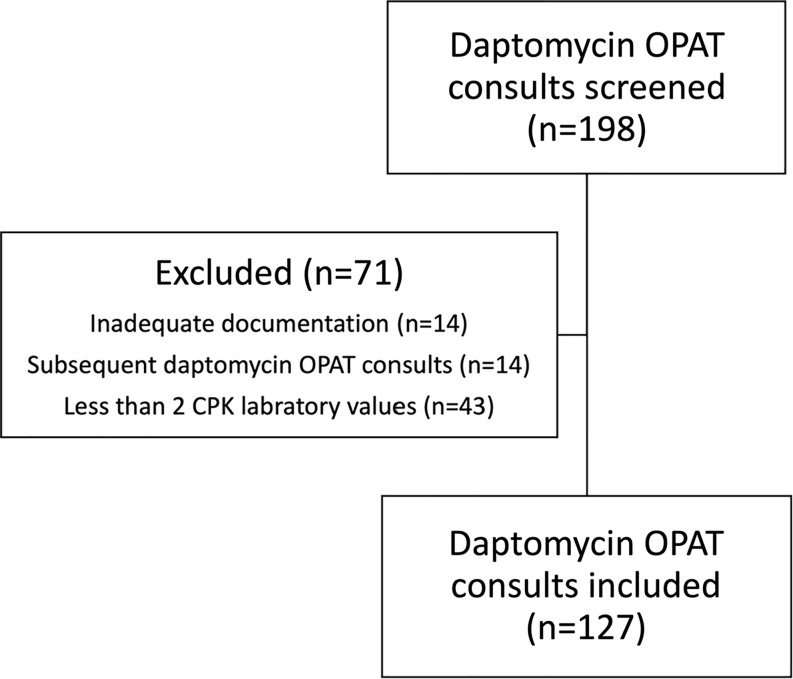
Daptomycin OPAT consults evaluated for inclusion.

There were 198 daptomycin OPAT consults screened for inclusion, of which 127 (65.8%) met criteria (Figure 1). The most common reason for exclusion was lack of follow up CPK values (n = 43, 22.3%). Most patients were male (n = 70, 55.1%), and the median age was 56 years (IQR 46–63). The median BMI was 28.2 kg/m^2^ (IQR 24–34.8) and 44.1% of patients (n = 56) had a BMI greater than 30 kg/m^2^. Only 29.1% of patients (n = 37) were on concomitant statin therapy with atorvastatin being the most common (n = 24,18.9%). Baseline characteristics are listed in Table 1.

**Table 1.** Baseline characteristics

Characteristics	Total (n = 127) n (%) or median (IQR)	CPK > 500 (n = 15) n (%) or median (IQR)	CPK < 500 (n = 112)n (%) or median (IQR)	*P* value*
Male Sex	70 (55.1)	12 (80)	58 (51.8)	.039
Age, years	56 (46–63)	58 (45–66)	55 (46–61.5)	.400
Actual body weight, kg	86.4 (68–102.7)	86 (78.6–110)	86.5 (66.8–102.5)	.604
Body Mass Index (BMI), kg/m^2^	28.2 (24–34.8)	27.1 (25.1–34.8)	28.6 (23.8–34.2)	.872
Social History: Alcohol Cannabis Methamphetamines	31 (24.4) 17 (13.4) 4 (3.1)	1 (6.7) 2 (13.3) 0 (0)	30 (26.8) 15 (13.4) 4 (3.6)	.115 1 1
Comorbidities: Obesity (BMI > 30 kg/m^2^) Chronic Kidney Disease Cirrhosis Diabetes Mellitus Coronary Artery Disease Cerebrovascular Disease Congestive Heart Failure Hypothyroidism Disease Immunosuppressed^#^ Cancer (Actively Treating) Seizure disorder	56 (44.1) 20 (15.7) 7 (5.5) 52 (40.9) 16 (12.6) 4 (3.1) 11 (8.7) 20 (15.7) 16 (12.6) 16 (12.6) 13 (10.2)	6 (40) 2 (13.3) 0 (0) 9 (60) 2 (13.3) 2 (13.3) 1 (6.7) 1 (6.7) 2 (13.3) 3 (20) 0 (0)	50 (44.6) 18 (16.1) 7 (6.3) 43 (38.4) 14 (12.5) 2 (1.8) 10 (8.9) 19 (17) 14 (12.5) 13 (11.6) 13 (11.6)	.734 1 1 .11 1 .068 1 .463 1 .403 .362
Medications: Statin Atorvastatin Pravastatin Rosuvastatin Simvastatin Lovastatin Antihistamine Fibrate Chemotherapy/Biologic Therapy Angiotensin Receptor Blocker (ARB)	37 (29.1) 24 (18.9) 6 (4.7) 2 (1.6) 4 (3.1) 1 (.8) 32 (25.2) 2 (1.6) 13 (10.2) 17 (13.4)	6 (40) 5 (33.3) 0 (0) 0 (0) 1 (6.7) 0 (0) 3 (20) 2 (13.3) 3 (20) 4 (26.7)	31 (27.7) 19 (17) 6 (5.4) 2 (1.8) 3 (2.7) 1 (.9) 29 (25.9) 0 (0) 10 (8.9) 13 (11.6)	.368.759 .013 .183 .118
Serum creatinine (SCr), mg/dL	.9 (.6–1.3), n = 119	1.2 (.9–2.2)	.8 (.6–1.2), n = 104	.008
Creatine phosphokinase (CPK), U/L	38 (24.5–73), n = 116	57 (30–156)	36 (23–72), n = 101	.073
Alanine aminotransferase (ALT), U/L	18.5 (12–28), n = 86	19 (12–33), n = 11	18 (12–28), n = 75	.99
Aspartate aminotransferase (AST), U/L	21.5 (18–31), n = 86	23 (19–34), n = 11	21 (17–29), n = 75	.415
Length of stay, days	9 (5–14)	9 (5–14)	9 (5.5–14)	.949

* comparing patients who experienced the primary outcome, CPK > 500 U/L, and patients who did not experience the primary outcome, CPK < 500 U/L.

# immunosuppressed16 is defined as: High dose steroids—prednisone ≥ 2 mg/kg or ≥20 mg daily for at least 14 days, Receiving biologic agents in the preceding 6 months, Received a solid organ transplant, Hematopoietic cell transplantation (HCT) or chimeric antigen receptor (CAR) T-cell therapy within one year, or HCT at any time point on active graft versus host disease (GVHD) treatment, Received cancer chemotherapy within 6 months, Congenital immunodeficiency, or Human immunodeficiency virus (HIV) with a CD4 count ≤ 200 cells/μL.

The median daptomycin daily dose was 7.4 mg/kg (IQR 6.1–8.1) actual body weight and 8 mg/kg (IQR 7.3–8.4) adjusted body weight (Table 2). The most common infections were bone/joint infections (73.2%, n = 93), complicated bacteremia (22.1%, n = 28), and skin and skin structure infections (29.1%, n = 37, Table 2). Methicillin-resistant *Staphylococcus aureus* (MRSA) (43.3%, n = 55) was the most common pathogen isolated, whereas 16.5% of the patients did not have a microbiological diagnosis. Overall, patients received daptomycin for a median 37 days (IQR 21–44) and ranging from 6 to 118 days. The median inpatient daptomycin duration was 4 days (IQR 2–6.5) and 35 days outpatient (IQR 25–42). Patients had a median of 5 CPK labs drawn during therapy (IQR 3–6).

**Table 2.** Daptomycin and infection characteristics

Characteristics	Total (n = 127) n (%) or median (IQR)	CPK > 500 (n = 15) n (%) or median (IQR)	CPK < 500 (n = 112) n (%) or median (IQR)	*P* value*
Daptomycin Dose, mg	600 (500–700)	650 (500–700)	600 (500–700)	.791
Daptomycin Daily Dose, mg/kg^ Actual body weight Adjusted body weight	8 (7.5–8.4) 7.4 (6.1–8.1) 8 (7.3–8.4),	7.9 (7.3–8.3) 7.1 (6.1–7.9) 7.9 (7.9–8),	8 (7.5–8.4) 7.4 (6–8.1) 8 (6.9–8.4),	.234 .453 .989
Duration of Daptomycin, days Inpatient Daptomycin Duration, days Outpatient Daptomycin Duration, days	37 (21–44) 4 (2–6.5), n = 100 35 (25–42)	15 (14–26) 4 (2–5), n = 13 13 (9–25)	38 (28–47) 4 (2–7), n = 87 36 (28–42.5)	<.001 .345 <.001
Indication: Bone/joint infections Skin and Soft Tissue Infection (SSTI) Complicated Bacteremia Endocarditis Urinary Tract Intra-abdominal Other	93 (73.2) 37 (29.1) 28 (22.1) 5(3.9) 1 (.8) 6 (4.7) 3 (2.4)	10 (66.7) 2 (13.3) 5 (33.3) 1(6.7) 0 0 0	83 (74.1) 35 (31.3) 23 (20.5) 4 (3.9) 1 (.9) 6 (5.4) 3 (2.9)	.544 .228 .319 .472 1 1 NA
Organism: Methicillin-resistant *S*. *aureus* (MRSA) Methicillin-susceptible *S*. *aureus* (MSSA) Coagulase-negative *Staphylococcus* (CoNS) * Enterococcus faecalis* Vancomycin-resistant *Enterococcus faecium* (VRE) * Streptococcus sp*. Culture negative Cultures not collected	55 (43.3) 4 (3.1) 24 (18.9) 8 (6.3) 10 (7.9) 11 (8.7) 21 (16.5) 8 (6.3)	6 (40) 0 (0) 6 (40) 2 (13.3) 2 (13.3) 2 (13.3) 1 (6.7) 2 (13.3)	49 (43.8) 4 (3.6) 18 (16.1) 6 (5.4) 8 (7.1) 9 (8) 20 (17.9) 6 (5.4)	.783 1 .034 .24 .335 .618 .463 .24

* comparing patients who experienced the primary outcome, CPK > 500 U/L, and patients who did not experience the primary outcome, CPK < 500 U/L.

^ calculated using actual body weight for BMI < 30 kg/m^2^ (n = 71) and adjusted body weight for BMI > 30 kg/m2 (n = 56) (15).

### Outcomes

Fifteen patients (11.8%) experienced an elevated CPK greater than 500 U/L within a median 13 days from the first dose of daptomycin (IQR 9–16, Table 3). Patients who had a CPK greater than 500 U/L on daptomycin had a higher baseline serum creatinine (1.2 mg/dL vs .8 mg/dL, *P* = .0081) and were overwhelmingly male (n = 12, 80%). Twelve patients (9.4%) had CPK greater than 1,000 U/L, and eight patients (6.3%) had CPK greater than 2,000 U/L. Overall, there were only 18 (14.2%) reported adverse events with significantly more events occurring when the CPK was greater than 500 U/L (*P* < .0001). Seven patients had myopathy or myalgia (5.5%), five patients had rhabdomyolysis (3.9%), four patients (3.1%) experienced asymptomatic CPK elevation, and two patients (1.6%) had infusion site reactions without an elevated CPK. Twelve patients (80%) with a CPK greater than 500 U/L discontinued daptomycin and five patients (33.3%) had documented rhabdomyolysis with four (26.7%) requiring hospitalization for rhabdomyolysis. On the other hand, four patients with a CPK < 500 U/L (3.6%) discontinued daptomycin (*P* < .0001 compared to CPK>500 U/L), three of whom reported myopathy or myalgia, none required hospitalization. Of the five patients with rhabdomyolysis, all had CPK>1,000 U/L and four (80%) had CPK>2,000 U/L. Specific daptomycin-related outcomes are listed in Table 3. Concomitant use of a statin (40%, n = 6 vs 27.7%, n = 31, *P* = .368) was not a risk factor for CPK> 500 U/L. Higher serum creatinine values (1.2 vs .8 mg/dL, *P* = .008) was associated with CPK > 500 U/L in univariate analysis. Independent predictors of CPK > 500 U/L on multivariable logistic regression included male sex (OR, 4.2 [95% CI, 1.05–16.61]; *P* = .0424) and cerebrovascular disease (OR, 11 [95% CI, 1.21–99.86]; *P* = .0332), but not serum creatinine (Table 4).

**Table 3.** Daptomycin adverse event outcomes

Outcome	Total (n = 127) n (%) or med (IQR)	CPK > 500 (n = 15) n (%) or med (IQR)	CPK < 500 (n = 112) n (%) or med (IQR)	*P* value*
Primary				
CPK>500 U/L	15 (11.8)			
Time-to-event, days	13 (9–16)			
Secondary				
Incidence of complications of daptomycin^#^	20 (15.7)			
Time-to-event, days	13.5 (8–19.5)			
CPK>1,000 U/L	12 (9.4)			
Time-to-event, days	13.5 (10.5–18)			
CPK>2,000 U/L	8 (6.3)			
Time-to-event, days	14 (10.5–22)			
Development of myopathy/myalgia	7 (5.5)	4 (26.7)	3 (2.7)	.004
Time-to-event, days	14 (7–21)	14 (10.5–14.5)	21 (7–36)	.472
Development of rhabdomyolysis	5 (3.9)	5 (33.3)	0 (0)	<.001
Time-to-event, days	14 (6–14)	14 (6–14)	NA	
Daptomycin discontinuation	16 (12.6)	12 (80)	4 (3.6)	<.001
Time-to-event, days	14 (7–18)	14 (10.5–16.5)	17 (5.5–31.5)	.951
Daptomycin-related hospital readmission	4 (3.1)	4 (26.7)	0 (0)	<.001
Time-to-event, days	10 (6–17)	10 (6–17)	NA	

* comparing patients who experienced the primary outcome, CPK > 500 U/L, and patients who did not experience the primary outcome, CPK < 500 U/L.

# composite of CPK > 2,000 U/L, myopathy/myalgia, rhabdomyolysis, discontinuation of daptomycin, or hospital readmission for a daptomycin-related adverse event.

**Table 4.** Multivariable logistic regression for primary outcome of CPK>500 U/L

Variable	Comparator	Reference	Individual OR (95% CI)	*P* value	Adjusted OR (95% CI)	*P* value
Sex	Male, 55%	Female, 45%	3.72 (1.00–13.91)	.051	4.18 (1.05–16.61)	.042
Cerebrovascular Disease	Yes, 3%	No, 97%	8.46 (1.10–65.22)	.041	11.00 (1.21–99.86)	.033
Daptomycin Dose, mg	Any + 1 unit		.76 (.47–1.22)	.249		
Baseline Serum creatinine (SCr), mg/dL^#^	Any + 1 unit		1.49 (.93–2.41)	.100		
Baseline creatine phosphokinase (CPK), U/L^#^	Any + 1 unit		1.00 (1.00–1.01)	.072		
Body Mass Index (BMI)>30	Yes, 44%	No, 56%	.83 (.28–2.48)	.734		
Social History of Alcohol	Yes, 24%	No, 76%	.20 (.03–1.55)	.122		
Coag MRSE	Yes, 9%	No, 91%	3.48 (1.1–10.99)	.033		

*Note*: Overall sample percentages were added to variable comparator and reference values for convenience.

OR, Odds Ratio; CI, Confidence Interval; MRSE, Methicillin-resistant Staphylococcus epidermidis.

Variables of primary interest in the initial logistic regression model and reported unadjusted were daptomycin dose, SCr, CPK, BMI, social history of alcohol, and MRSE. Subsequent exploratory model using backward selection with *P* < .10 inclusion included these variables with sex, cerebrovascular disease, diabetes mellitus, hypothyroidism, cancer with active treatment, any statin use, any antihistamine use, E. faecalis, and osteomyelitis, SSTI, complicated bacteremia, or prosthetic joint infections.

# For eight patients with missing data, sample median values of .88 and 38 were imputed for Scr and CK, respectively.

## Discussion

This study aimed to evaluate the incidence of CPK elevation and risk factors associated with adverse effects when using daptomycin for OPAT. Our study showed an incidence of 11.8% for CPK elevations greater than 500 U/L in patients who received daptomycin at a median 8 mg/kg adjusted body weight for a median duration of 35 days. Male sex and cerebrovascular disease were identified as independent risk factors for CPK elevation.

Previous studies reported elevated CPK rates ranging from 2% to 12%, which aligns with our study results.^4–6,13,17,18^ However, the definition of CPK elevation is heterogeneous across published studies, with some defining it as any elevation greater than the upper limit of normal (200 U/L),^13,16^ over 500 U/L,^12, 22^ or greater than five times the upper limit of normal (1,000 U/L).^11,12,14,15^ Our patient population received higher doses compared to other published cohorts, at a median of 8 mg/kg/dose versus. 6 mg/kg/dose, which could explain why our incidence of CPK elevation is on the higher side of the reported range in the literature .^13,17,18^ Furthermore, the average duration of daptomycin therapy in prior studies ranged between 12 and 25 days, ^5,11,13,17,18^ compared to a median of 37 days in our cohort. Our rate of rhabdomyolysis of 3.9% was higher than the 1% previously reported.^13^ Nevertheless, the majority of the studies^5,13,17^ reported a time to CPK elevation within the first two weeks of therapy which is consistent with these results. The time line from other studies showing elevation of CPK within the first 2 weeks of therapy supports weekly monitoring for patients on higher doses of daptomycin.

While univariate analysis showed a statistically significant higher baseline SCr in patients who experienced CPK>500 U/l compared to those who did not, our multivariable regression analysis found no association. Our study did not find that concomitant use of a statin was associated with CPK elevation. The literature is divided on this point with some studies showing concomitant use of a statin was associated with CPK elevation^12,13,15,16^ while other studies showing no increased risk.^11,14^ Our study also found an association between a history of cerebrovascular disease or male sex and an elevated CPK which has not been noted in previous studies. While a prior cerebrovascular accident affected only four patients in our cohort, this should be interpreted cautiously and be hypothesis generating for future studies. With the large confidence interval for male sex, it should be evaluated in future studies. The patient characteristics associated with increased CPK elevations are not consistent in the literature, and recommendations for specific patient populations that need closer monitoring cannot be made at this time.

Daptomycin is a preferred therapy option for OPAT because it is well tolerated and dosed once daily. Compared to vancomycin for OPAT, there are fewer adverse events and patients can receive daptomycin for a longer duration before the onset of any events.^8^ Daptomycin is also associated with greater patient satisfaction with OPAT and with less disruption of their normal activities.^10^ With daptomycin being cheaper and easier to coordinate,^22–24^ the time of vancomycin for OPAT is fading.^25,26^With a time to increased CPK of 13 days (IQR 9–16), our study provides data to support the current recommendations of weekly CPK monitoring^7^ in a population that received higher doses for extended courses of daptomycin.

This study is limited by its single-center, retrospective design. Our institution used multiple EHRs at the time of data collection, which required additional vigilance for data reconciliation. Home medications were based on the patient’s discharge medication reconciliation note which may not have included a completely accurate list of medications, and past medical history was identified primarily through the physician’s initial admission note. By only including a patient’s first daptomycin consult, we might have missed subsequent CPK elevations and patient-specific factors contributing to elevations. Our patient labs are sent from numerous outside facilities without standard reference ranges, so we utilized 500 U/L as our definition of initial CPK elevation. There has been variation in the literature of what CPK value should be used for daptomycin safety from 200 to 2,000 U/L as discussed earlier .^4,11,13,17,18^ The cut off value may have affected our results and impacted the ability to determine patient-specific risk factors for elevations. Additionally, myopathy/myalgia and rhabdomyolysis were identified based on patient reports and physician documentation, so it is possible these numbers are under reported. Additional studies are needed to further evaluate risk factors for patients receiving daptomycin as outpatient parenteral antimicrobial therapy.

High-dose daptomycin (8 mg/kg) once daily given for 5 weeks in the OPAT setting had similar CPK elevations to previous studies with lower doses and shorter durations. The time to CPK elevation was a median of 13 days (IQR 9–16), supporting the current guidance of weekly monitoring. Overall, this study provides evidence that the current guidance is appropriate to identify initial elevations and adjust doses prior to the onset of musculoskeletal adverse events requiring therapy discontinuation.
